# Effects of aliskiren on mortality, cardiovascular outcomes and adverse events in patients with diabetes and cardiovascular disease or risk: A systematic review and meta-analysis of 13,395 patients

**DOI:** 10.1177/1479164117715854

**Published:** 2017-08-27

**Authors:** Sean L Zheng, Alistair J Roddick, Salma Ayis

**Affiliations:** 1 Imperial College Healthcare NHS Trust, London, UK; 2 British Heart Foundation Centre of Research Excellence, Cardiovascular Division, King’s College Hospital, London, UK; 3 Faculty of Life Sciences & Medicine, King’s College London, London, UK; 4 Department of Primary Care and Public Health Sciences, King’s College London, London, UK

**Keywords:** Aliskiren, direct renin inhibitor, renin–angiotensin inhibition, diabetes, systematic review, meta-analysis

## Abstract

**Background:**

Aliskiren was shown to increase adverse events in patients with diabetes and concomitant renin–angiotensin blockade. We aim to investigate the efficacy and safety of aliskiren in patients with diabetes and increased cardiovascular risk or established cardiovascular disease.

**Methods:**

MEDLINE and Embase were searched for prospective studies comparing addition of aliskiren to standard medical therapy in patients with diabetes and cardiovascular disease, or ⩾1 additional cardiovascular risk factor (hypertension, abnormal lipid profile, microalbuminuria/proteinuria, chronic kidney disease). Relative risk for efficacy (all-cause mortality, combined cardiovascular mortality and hospitalisation) and safety (hyperkalaemia, hypotension, renal impairment) outcomes was calculated.

**Results:**

Of 2151 studies identified in the search, seven studies enrolling 13,395 patients were included. Aliskiren had no effect on all-cause mortality (relative risk: 1.05, 95% confidence interval: 0.90 to 1.24, *p* = 0.53), or combined cardiovascular mortality or heart failure hospitalisation (relative risk: 1.07, 95% confidence interval: 0.81 to 1.40, *p* = 0.64). Patients receiving aliskiren had a greater risk of developing hyperkalaemia (relative risk: 1.32, 95% confidence interval: 1.14 to 1.53, *p* = 0.0003) and renal impairment (relative risk: 1.15, 95% confidence interval: 1.02 to 1.30, *p* = 0.03), but not hypotension.

**Conclusion:**

Patients with diabetes and cardiovascular disease or cardiovascular risk do not benefit from the addition of aliskiren to standard medical therapy. Detrimental safety profile in pooled analysis supports current warnings.

## Background

The direct renin inhibitor (DRI) aliskiren has been proposed as an alternative to angiotensin II-converting enzyme inhibitor (ACEi) or angiotensin II receptor blocker (ARB) therapy in the management of cardiovascular morbidity and mortality in diabetes mellitus,^1^ with similar antihypertensive efficacy to ACEi or ARB.^2–5^ However, large clinical trials evaluating cardiovascular clinical endpoints have failed to demonstrate non-inferiority with aliskiren compared to ACEi or ARB treatment and have identified potential safety concerns in diabetic patients when used in addition with ACEi or ARB.^6,7^

The aim of this meta-analysis is to investigate the efficacy and safety of aliskiren use in addition with background medical therapy in patients with diabetes and high cardiovascular risk or established cardiovascular disease.

## Methods

This article is written in accordance with the Preferred Reporting Items for Systematic Reviews and Meta-Analysis guidelines.^8^ No published protocol for this systematic review and meta-analysis exists.

### Data sources and search strategy

A systematic search of MEDLINE and Embase databases was performed on 3 October 2016 using the terms *Aliskiren*, *Tekturna*, *Rasilez*, *SPP100*, *renin inhibitor* and *Diabetes*. Results were filtered from 1 January 2000. The reference lists of included studies and reviews were hand-searched for additional articles. The Novartis clinical trials database^9^ was searched for additional data of completed trials.

### Study selection criteria

We included prospective studies that enrolled patients with diabetes and established or history of cardiovascular disease, or at least one cardiovascular risk factor (study-defined hypertension, raised low-density lipoprotein, reduced high-density lipoprotein, microalbuminuria, proteinuria or chronic kidney disease). Studies were required to compare aliskiren dual therapy with ACEi or ARB, with either placebo or ACEi/ARB monotherapy. Included studies were required to report all-cause mortality stratified by diabetes status. To assess the long-term efficacy, studies with follow-up (mean/median) of ⩾6 months were included.

### Data extraction and quality assessment

Study selection, data extraction and quality assessment were performed independently by two authors (S.L.Z., A.R.). After removal of duplicates, title and abstracts were screened for relevance, with full texts of remaining results assessed for inclusion based on pre-determined inclusion criteria. Inclusion required agreement between reviewers (S.L.Z., A.R.). The data extracted from each report included general study characteristics (study name, primary investigator, year of publication, median or mean duration of follow-up, inclusion and exclusion criteria), participant characteristics (number, age, gender, cardiovascular co-morbidities – hypertension, heart failure, previous myocardial infarction, concomitant use of ACEi and/or ARB), outcome data [hazard risk, risk or odds ratios with 95% confidence intervals (CIs), and absolute numbers] and adverse events (study-defined hyperkalaemia, hypotension and renal impairment).

Study corresponding authors were contacted for additional information where required.

The Cochrane Collaboration risk of bias tool was used to assess risk of bias. All studies had low risk of bias, with no impact on data synthesis.

### Statistical analysis

Primary efficacy outcome was all-cause mortality. Secondary efficacy outcome was combined cardiovascular mortality and heart failure hospitalisation. Primary safety outcomes were hyperkalaemia, hypotension and renal impairment. Relative risk (RR) was calculated from raw published study data. Review Manager 5.3 was used for statistical analysis.^10^ Mantel–Haenszel method was used to calculate estimates, CIs and *p*-values. Random-effects models were used to summarise data. *p*-values for heterogeneity were calculated using chi-square test.

## Results

### Study selection and baseline characteristics

**Figure 1.**
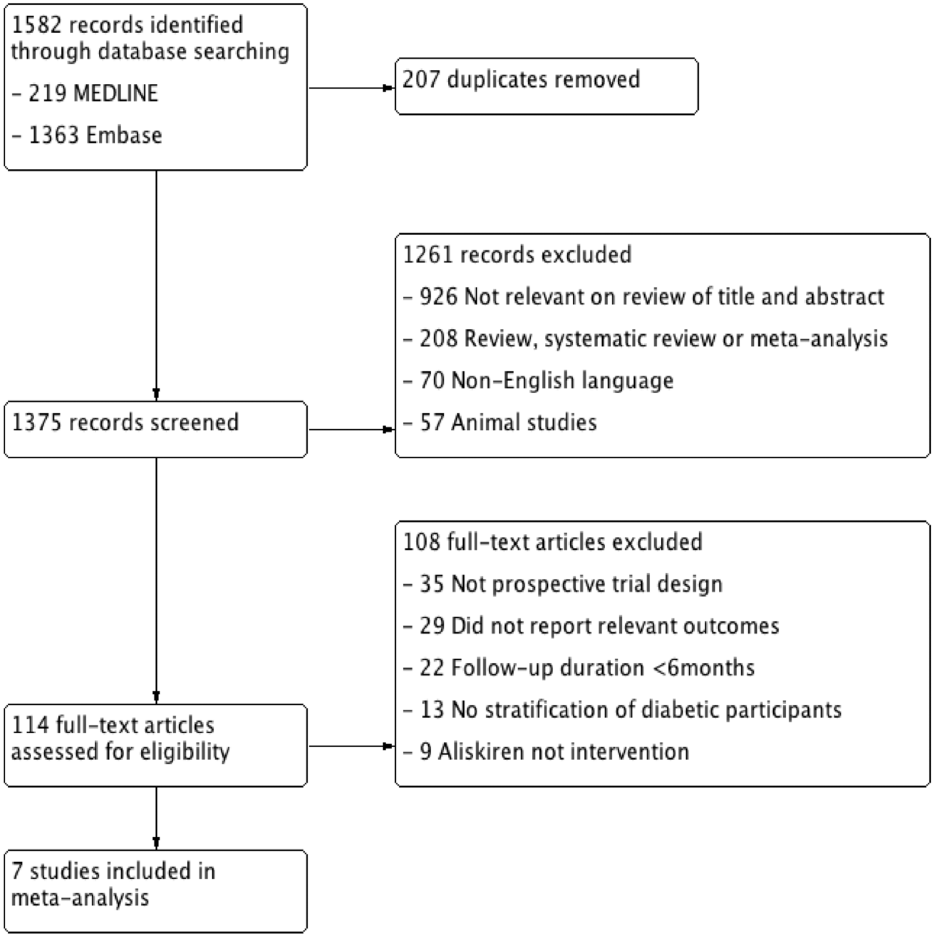
Study flow chart.

The search identified 2150 articles. Seven were included for meta-analysis (Figure 1 – study flow chart), enrolling 13,395 patients with diabetes (Table 1 – baseline characteristics). The participants were followed up for a mean of 24.8 months, totalling 27,683 patient-years follow-up. There were five randomised controlled trials (RCTs)^11–16^ and one observational study.^17^ The largest trials were Aliskiren Trial in Type 2 Diabetes Using Cardiorenal Endpoints (ALTITUDE); (8552 participants),^12^ the 3A registry (1936)^17^ and Aliskiren Trial to Minimize Outcomes in Patients with Heart Failure (ATMOSPHERE); (1317).^16^ Two RCTs enrolled participants with diabetes and high cardiovascular risk (Aliskiren in the Evaluation of Proteinuria in Diabetes (AVOID), ALTITUDE, *n* = 9151), four RCTs enrolled participants with diabetes and established cardiovascular disease [Aliskiren Study in Post-MI Patients to Reduce Remodelling (ASPIRE), Aliskiren Trial on Acute Heart Failure Outcomes (ASTRONAUT), Aliskiren Quantitative Atherosclerosis Regression Intravascular Ultrasound Study (AQUARIUS), ATMOSPHERE, n = 2935] and one observational study enrolled participants with hypertension (3A Registry, *n* = 3038).

**Table 1.** Study characteristics of included trials.

Trial, author	Year	Trial type	Inclusion criteria	Primary outcomes	Intervention	Control	Follow-up (m)	Total	Intervention	Control	Male	Age	Htn	HF	Prior MI	ACEi	ARB	RAS
AVOID, Parving^11^	2008	RCT	Hypertension, diabetes, nephropathy	Reduction in ACR	Aliskiren + Losartan	Losartan	6	599	301	298	427 (71%)	61	599 (100%)	NR	34 (6%)	0 (0%)	599 (100%)	599 (100%)
ALTITUDE, Parving^12^	2012	RCT	Diabetes, micro or macroalbuminuria, CVD	CV mortality, cardiac arrest, nonfatal MI/stroke, HFH, renal outcomes	Aliskiren + SMT	Placebo, SMT	32.9	8552	4274	4278	5826 (68%)	65	NR	872 (10%)	1424 (17%)	3787 (44%)	4796 (56%)	8552 (100%)
ASPIRE, Shah^13^	2012	RCT	Post-acute MI with HF	CV mortality, HFH, MI, stroke, cardiac arrest	Aliskiren + SMT	Placebo, SMT	8.3	214	112	102	175 (82%)	62	146 (68%)	12 (6%)	214 (100%)	189 (88%)	24 (11%)	213 (100%)
ASTRONAUT, Maggioni^14^	2013	RCT	HF, LVEF <40%, raised biomarkers	CV mortality, HFH	Aliskiren + SMT	Placebo, SMT	12	662	319	343	508 (77%)	66	542 (82%)	662 (100%)	NR	414 (61%)	152 (23%)	566 (84%)
AQUARIUS, Puri^15^	2015	RCT	Coronary artery stenosis, CV risk factors	Change in % atheroma volume	Aliskiren + SMT	Placebo, SMT	24	115	55	60	90 (78%)	61	103 (90%)	NR	33 (29%)	71 (62%)	29 (25%)	100 (87%)
ATMOSPHERE, McMurray^16^	2016	RCT	Symptomatic HF, LVEF <35%, raised biomarkers	CV mortality, HFH	Aliskiren	Enalapril	24.1	1317	665	652	1027 (78%)	64	988 (75%)	856 (65%)	632 (48%)	1317 (100%)	0 (0%)	1317 (100%)
3A Registry, Kistner^17^	2016	POS	Hypertension	NA	Aliskiren + ACEi/ARB	ACEi/ARB	12	1936	1381	555	1137 (59%)	67	1936 (100%)	447 (23%)	NR	–	–	1936 (100%)

ALTITUDE: Aliskiren Trial in Type 2 Diabetes Using Cardiorenal Endpoints; ATMOSPHERE: Aliskiren Trial to Minimize Outcomes in Patients with Heart Failure; ASPIRE: Aliskiren Study in Post-MI Patients to Reduce Remodelling; ASTRONAUT: Aliskiren Trial on Acute Heart Failure Outcomes; AQUARIUS: Aliskiren Quantitative Atherosclerosis Regression Intravascular Ultrasound Study; RCT: randomised controlled trial; POS: prospective observational study; CV: cardiovascular; HF: heart failure; LVEF: left ventricular ejection fraction; MI: myocardial infarction; ACEi: angiotensin-converting enzyme inhibitor; ARB: angiotensin receptor blocker; RAS: renin–angiotensin system blocker; ACR: albumin-to-creatinine ratio; HFH: hospitalisation for heart failure; MI: myocardial infarction; CVD: cardiovascular disease; SMT: standard medical therapy (includes ACEi and ARB); NR: not recorded; Htn: hypertension.

### Effect of aliskiren on outcomes

**Figure 2.**
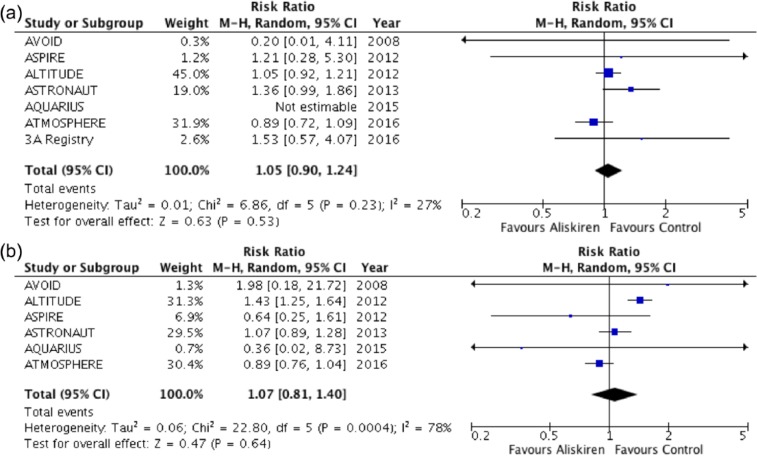
Forest plot of (a) all-cause mortality and (b) combined cardiovascular mortality and hospitalisation for heart failure.

Aliskiren was associated with the same risk of death compared with controls (RR: 1.05, 95% CI: 0.90 to 1.24, *p* = 0.53, *I*^2^ = 27%; Figure 2(a)). There was no difference in combined cardiovascular mortality and heart failure hospitalisation between aliskiren and controls (RR: 1.07, 95% CI: 0.81 to 1.40, *p* = 0.64, *I*^2^ = 78%; Figure 2(b)).

Among 2308 patients with established cardiovascular disease, aliskiren was not associated with reductions in all-cause mortality (RR: 1.08, 95% CI: 0.76 to 1.54, *p* = 0.66) or combined cardiovascular mortality and heart failure hospitalisation (RR: 0.96, 95% CI: 0.83 to 1.09, *p* = 0.51) compared with controls (Supplementary Figure 1).

### Effect of aliskiren on blood pressure and urinary albumin-to-creatinine ratio

Three trials reported change in blood pressure between aliskiren and control groups, all of which favoured the addition of aliskiren. Two trials reported on change in urinary albumin-to-creatinine ratio, again both in favour of aliskiren (Table 2).

**Table 2.** Changes in urinary albumin-to-creatinine ratio, blood pressure (difference in change between aliskiren and control group) and discontinuations due to adverse events in randomised controlled trials.

Trial	Baseline ACR (aliskiren group)	% change in urine ACR	Baseline sitting blood pressure (aliskiren group)	Change in blood pressure (aliskiren − control)	Discontinuations due to adverse event – aliskiren group	Discontinuations due to adverse event – control group
AVOID	513	−18%	135/78 mmHg	−2/1 mmHg	17 (5.6%)	19 (6.4%)
ALTITUDE	206	−16%	137/74 mmHg	−1.9/1 mmHg	563 (13.2%)	437 (10.2%)
ASPIRE	NR	NR	NR	NR	NR	NR
ASTRONAUT	NR	NR	NR	NR	NR	NR
AQUARIUS	NR	NR	132/77 mmHg	−3/0.6 mmHg	NR	NR
ATMOSPHERE	NR	NR	127 mmHg (SBP)	NR	NR	NR

ALTITUDE: Aliskiren Trial in Type 2 Diabetes Using Cardiorenal Endpoints; ATMOSPHERE: Aliskiren Trial to Minimize Outcomes in Patients with Heart Failure; ASPIRE: Aliskiren Study in Post-MI Patients to Reduce Remodelling; ASTRONAUT: Aliskiren Trial on Acute Heart Failure Outcomes; AQUARIUS: Aliskiren Quantitative Atherosclerosis Regression Intravascular Ultrasound Study; ACR: albumin-to-creatinine ratio; NR: not reported; SBP: systolic blood pressure.

### Adverse events

**Figure 3.**
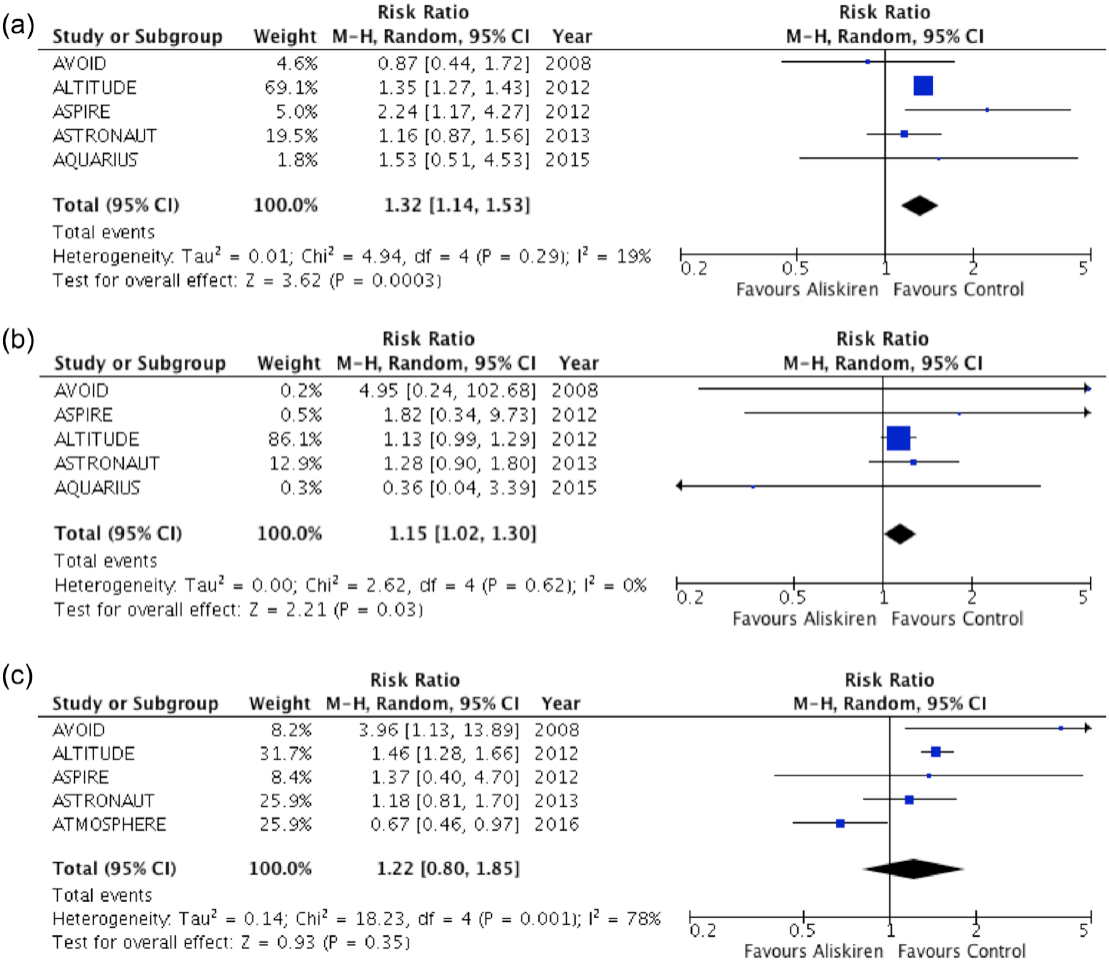
Forest plot of adverse events: (a) hyperkalaemia, (b) renal impairment and (c) hypotension.

Rates of hyperkalaemia, renal impairment and hypotension were reported in five, five and four RCTs, respectively. Patients receiving aliskiren had greater risk of developing hyperkalaemia [RR: 1.32, 95% CI: 1.14 to 1.53, *p* = 0.0003, absolute risk reduction (ARR): 6%] and renal impairment (pooled RR: 1.15, 95% CI: 1.02 to 1.30, *p* = 0.03, ARR 1%) compared with controls (Figure 3(a) and (b)). There was no increase in risk of developing hypotension in patients with diabetes given aliskiren (RR: 1.22, 95% CI: 0.80 to 1.85, *p* = 0.035; Figure 3(c)). Study withdrawal in RCTs due to adverse events was only reported in two trials (Table 2).

### Study quality and risk of publication bias

All RCTs had low overall risk of bias, and funnel plot showed little evidence of publication bias (Supplementary Figures 2 and 3).

## Discussion

The findings of this meta-analysis demonstrate that aliskiren has no effect on all-cause mortality or combined cardiovascular mortality and heart failure hospitalisation in patients with diabetes and cardiovascular risk or established disease. Pooled analysis demonstrated an increased risk of hyperkalaemia and renal impairment with aliskiren.

This meta-analysis is the first to examine the efficacy and safety of aliskiren in trial patients with high-risk diabetes, a group where renin–angiotensin system (RAS) inhibition reduces cardiovascular outcomes.^18^ We compare use of aliskiren with placebo or single ACEi/ARB in patients with diabetes. There were high rates of concomitant ACEi or ARB use in trial participants (85–100% in individual trials, and 99.1% in the pooled trial population). Our findings can therefore be cautiously interpreted as an increased risk with dual RAS blockade using aliskiren for hyperkalaemia and renal impairment of 32% (population risk ranges: 14%–53%) and 15% (population risk ranges: 2%–30%) respectively. This supports the US Food and Drug Administration and European Medicines Agency safety announcements warning against the use of aliskiren in combination with ACEi or ARB in patients with diabetes.

Dual RAS blockade in diabetic patients with high cardiovascular risk does not improve outcomes and is associated with increased adverse events,^19^ a conclusion that is extended to the use of aliskiren and supported by this meta-analysis. It remains to be seen whether aliskiren as monotherapy is of benefit, and whether it serves as an alternative in patients intolerant of ACEi and ARB. In meta-analysis of blood pressure reduction, aliskiren showed superior efficacy over ACEi and equivalence with ARB.^20^ Current evidence suggests that in diabetic patients without specific indications for RAS blockade, RAS blockade was not superior in improving cardiovascular outcomes compared with alternative blood pressure–reducing therapies.^21^

### Study limitations

Limitations of this study include those inherent in meta-analyses, which are driven primarily by availability and accessibility of data. Adverse events were trial defined, and so conclusions on increased risk of these are not with universal definitions. We are unable to comment on whether there may be benefit of aliskiren over other RAS blockers stratified by cardiovascular diagnosis (e.g. heart failure, post-myocardial infarction or hypertension) due to inability to extract relevant data. While subgroup data from ATMOSPHERE suggested benefit of aliskiren in patients with diabetes and heart failure (HR: 0.87, Upper confidence interval (UCI): 1.09), pooled data from the three studies that enrolled heart failure patients showed no effect (data not presented).

In conclusion, in patients with diabetes and cardiovascular disease or raised cardiovascular risk, the addition of aliskiren has no effect on all-cause mortality and combined cardiovascular mortality or heart failure hospitalisation compared with control treatment. There is an increased risk of hyperkalaemia and renal impairment with the use of aliskiren in addition to baseline ACEi and ARB therapy. This study supports current warnings against aliskiren use in patients with diabetes.

## Supplementary Material

Supplementary material
